# Depression and Anxiety Among Arab Individuals in the United States: A Meta-analysis

**DOI:** 10.1007/s10903-024-01648-9

**Published:** 2024-11-27

**Authors:** Shaimaa Mosad El-Refaay, Christina Kenny, Sandra Weiss

**Affiliations:** 1 School of Nursing, UCSF, 2 Koret Way Rm 411Y, San Francisco, CA 94143 USA; 2 Division of Geriatrics, School of Nursing, UCSF, San Francisco, USA; 3 Department of Community Health Systems, Stress and Depression Lab, University of California, San Francisco, USA

**Keywords:** Arab, Anxiety, Depression, Immigrants, Refugees

## Abstract

Emerging research suggests that the increasing population of Arab immigrants and refugees living in the United States (U.S.) has a greater risk for depression and anxiety than other groups. The purpose of this meta-analysis was to estimate the prevalence of depression and anxiety for Arabs in the U.S., to examine the moderating effects of key demographic variables (gender, immigration status, ethnicity) and study characteristics (research design and quality) on the prevalence of anxiety and depression, and to evaluate the direct effects of additional demographic and study characteristics on prevalence rates. Using guidelines from the Preferred Reporting Items for Systematic Review and Meta-Analysis Protocols (PRISMA-P), we searched PubMed, Embase, CINHAL, ProQuest, and the Web of Science databases for articles published between January 2000 and March 2023. Two reviewers each individually extracted the articles’ data and evaluated the reported study’s methodological quality using a well-established checklist. Statistical analyses consisted of random effects models to estimate the pooled prevalence of depression and anxiety across all studies, separate random effects models for distinct demographic (e.g., immigration status) and methodologic (e.g., study quality) subgroups that might have unique prevalence rates, and a meta-regression to identify additional variables (e.g., age of individuals) that might influence prevalence rates. Our review included 17 studies with a total sample population size of 22, 558 participants. Results indicated that the estimated prevalence of depression overall was 48% (CI 34, 63%) and 58% (CI 33, 83%) for the prevalence of anxiety. Our subgroup analyses uncovered notable variations in prevalence rates based on gender composition, immigration status, ethnicity, and research quality while results of the meta-regressions demonstrated effects of publication year and age of the Arab individual on the estimated prevalence of depression and anxiety. The high prevalence rates for depression and anxiety identified through this meta-analysis are very concerning and underscore the urgent need to address mental health concerns in Arab communities. Results of subgroup and meta-regression analyses suggest that refugees, individuals from specific countries of origin, and younger Arabic persons may need particular attention by clinicians in screening for and treating their depression and anxiety. Our results also signify the need for further high-quality studies to more rigorously assess prevalence of these mental health problems, since we found evidence of potential publication bias and approximately one third of the studies in the review had less optimal methodological quality based on our evaluation. Ultimately, in-depth research regarding the causes of anxiety or depression among Arab individuals will be essential to the development of culturally competent interventions that may reduce their very high prevalence rates.

**Supplementary Information:**

The online version contains supplementary material available at 10.1007/s10903-024-01648-9.

## Introduction

The Arab population is quickly increasing in the United States [1]. According to the Arab American Institute, in 2016 almost 4 million Arabs of different source nationalities lived in the U.S., including those who were born overseas (first generation), U.S.-born descendants of immigrants (the 1.5 generation as well as the second generation), or U.S.-born to native-born parents (third generation and later) [2, 3]. Arab immigrants are typically defined as having at least one ancestor who lived in or immigrated from a nation of the Arab World, including the Arabian Peninsula, the Middle East, and Northern Africa [4, 5]. However, immigration is a unique concept defined in large part by whether the action was voluntary or not. For example, some individuals immigrated voluntarily to pursue better educational, social, or professional opportunities while others departed their country involuntarily (e.g., refugees escaping from oppression, war, or political intimidation) [5, 6].

Arab immigrants may experience many relocation stressors, including prejudice, racism, acculturation issues, social challenges, and language barriers [7]. Together with significant changes in lifestyle, such stressors increase the Arab American’s risk for mental problems such as anxiety or depression [7, 8]. Several authors have cited stressful living conditions and the socio-economic pressures of their new environments as significant sources of anxiety or depression for Arab immigrants in the U.S. [9, 10]. Researchers have also shown that Arab populations have greater rates of loneliness and isolation from society, of stress, of depression, and of anxiety, compared with other immigrant groups and compared to their host country’s population [4, 11, 12].

An increase in the number of displaced people or immigrants exhibiting mental health symptoms may exert significant additional pressure on the existing mental health services in the United States [13]. Depression and anxiety rank among the leading causes of disability-adjusted life years in the U.S. and are projected to become the second-most disabling conditions worldwide by 2030 [14]. Mental health issues, such as depression or anxiety, can profoundly affect an individual’s ability to concentrate, thereby undermining their capacity for social and productive integration [15]. However, Arab minorities are 23% less likely to undergo depression screening compared to non-Hispanic whites, suggesting that Arab individuals may be somewhat more likely to endure an undiagnosed depression or other mental health condition [11, 15]. Untreated mental conditions can result in a $300 billion annual loss in productivity for the U.S. [16].

The significance of studying the epidemiology of mental health symptoms in Arab populations residing in the U.S. increases in parallel to their increasing numbers [7]. A greater understanding of the prevalence of key mental health problems such as depression and anxiety may facilitate more active screening for these problems among Arab immigrants within both primary care and psychiatric care settings [17]. This information will also enable the development of interventions supporting Arab immigrants in managing their immigration-related stressors, ultimately promoting their mental health [7, 18]. Improved knowledge about the prevalence of mental disorders in Arab immigrants and refugees also has policy implications [13]. Designing more-suitable, evidence-based migration and social policies could substantially relieve the mental health burden within these populations [19]. For example, existing policies and services could be more linguistically and culturally appropriate to aid refugees to find work and to enable them to develop social networks in their new societies [19]. Identifying more culturally appropriate clinical interventions, along with altering extant policy to support improvements in the care provided, also depend on an accurate understanding of which groups of immigrants may be at greatest risk of mental health problems so that interventions can be developed and targeted specifically for these Arab individuals.

Several published systematic reviews have addressed the mental health epidemiology of immigrants in general, including mixed populations from varied countries [20–23]. For example, Foo et al. [22], in their meta-analysis, assessed the aggregate prevalence of depression among migrants worldwide and reported that the prevalence of depression among mixed immigrant groups was 14.8% (95% CI [8.4, 24.8]) of all depressive cases existing in the U.S. at that time. Blackmore et al. [21] evaluated the prevalence of mental illness reported by their source studies of child and adolescent refugee populations worldwide and found that the overall prevalence of depression was 13.81% (95% CI [5.96, 21.67]) with the prevalence of anxiety disorders estimated at 15.77% (95% CI [8.04, 23.50]). However, no systematic review has been focused on estimating the prevalence of mental health problems (aggregated or otherwise) in Arab communities in the U.S. To the best of our knowledge, there has been no systematic review with an explicit focus on the extent to which depression and anxiety (the most recognized immigration-related mental health problems) exist among Arab individuals living in the U.S. Thus, our aims in this review were: (1) to estimate the prevalence of depression and anxiety among Arab individuals in the U.S., and (2) to examine the moderating effects of three important demographic factors (gender, immigration status, and ethnicity) on the prevalence of anxiety or depression in our target population. To better understand the potential influence of study methodology on results, we also assessed the effect of study type and study quality on findings.

## Methods

We developed this research project’s study design using the PRISMA-P guidelines. These guidelines comprise a 17-item checklist by which researchers may evaluate systematically the accessible peer-reviewed literature and structure their report [24].

### Eligibility Criteria

Our eligibility criteria appear in Table 1. We used the PECO (T) (Population, Exposure, Comparison, Outcome, Study Type) method to describe our eligibility criteria [24]. U.S. Arab residents were our target population, including adults or children who immigrated into the U.S. (refugees, permanent visa holders, and others) or who were born in the U.S. and currently living there. Outcomes were the prevalence of depression or anxiety, and their symptom severity. We excluded articles focused on other mental health problems or on very specialized areas of depression or anxiety (e.g., panic disorder or post-partum depression).The type of study could be cross-sectional, case–control, or longitudinal research design. The study characteristics required for inclusion were as follows: full text, peer-reviewed, English-language only, and published between January 2000 and March 2022.

**Table 1:** PECOT table of study inclusion and exclusion criteria

PECO(T) criteria	Inclusion (eligible)	Exclusion (not eligible)
Population	Adult or child Arab immigrants (e.g. asylum seekers, refugees, permanent visa holders); U.S.- born Arabs living in the U.S	None
Exposure	Not applicable	Not applicable
Outcome	Prevalence of depression or anxiety or sufficient relevant data to estimate depression’s or anxiety’s prevalence	Investigations of the prevalence of general psychological distress or other mental health problems, (e.g., bipolar disorders, psychotic disorders) or of specialized types of depression or anxiety (e.g., panic disorder, post-partum depression)
	Severity of depression and anxiety symptoms, defined either by clinical diagnoses, structured clinical interviews, self-reported rating scales, or medical records	Investigations of the severity of other mental health problems (e.g., bipolar disorders, psychotic disorders) or specialized types of depression or anxiety (e.g., panic disorder, post-partum depression)
Study type	Cross-sectional, longitudinal, or case–control study designs	Case studies, case reports,
Report characteristics	Peer-reviewed, full-text articles written in English published between January 2000 and March 2022	Newspaper or magazine articles, conference papers, systematic reviews, papers reporting only a study’s protocol, or dissertations. Also, any peer-reviewed full-text English-language articles published prior to January 2000 or after March 2022

### Information Sources

We used the following electronic databases: PubMed, Psych INFO, EMBASE, CINHAL, and the Web of Science. In collaboration with a research librarian, we developed a comprehensive search strategy using both the medical subject heading (MeSH) terms and text words specific to each database.

### Search Strategy

In Appendix I, we show the search strategies created for the first four databases listed above. The research librarian assisted in formulating and applying the most effective combination of relevant search terms, including both text words and MeSH terms. Our strategy prioritized the following two primary conceptual areas: (1) depression or anxiety; and (2) our target population of Arab individuals living in the U.S. For the first concept, we included “depression,” “depressive disorder,” “anxiety,” “anxiety disorders”, “mental health” as our MeSH terms. For the second concept, we included the MeSH terms “Arabs,” “immigrants,” “transient or migrants”, “emigrants and immigrants” together with keywords such as “refugees”, “asylum seekers” or “Arab American”. We then combined all the developed search themes using the ‘AND’ Boolean operator. We also did not limit the search to specific study types (e.g., cross-sectional, or longitudinal) to avoid unintentionally ruling out potentially relevant studies.

### Data Production and Quality Checks

Our team developed screening questions to filter search results based on our eligibility criteria. A single reviewer (SE) first imported all the papers we had obtained from the electronic database searches into the reference management application (Endnote V. x9) we had selected for the first round of duplicate removal. SE then transferred the article dataset to the Covidence software package (a systematic review management application) for further duplicate removal. Any articles lacking either an abstract or the full text were excluded. SE next used Covidence to store the eligible articles and to extract the data needed for analysis. After all duplicates were removed, SE then transferred the data from each article from Covidence to Microsoft Excel 2017 to extract data from the text and to screen search results. Ch K double-checked all SE’s work to reduce errors involving duplicates. When there were disagreements between SE and Ch K, SW acted as the third reviewer. We did not contact the authors of a rejected article to obtain the items lacking from their paper because we had an adequate number of eligible articles.

## Selection Process

**Fig. 1:**
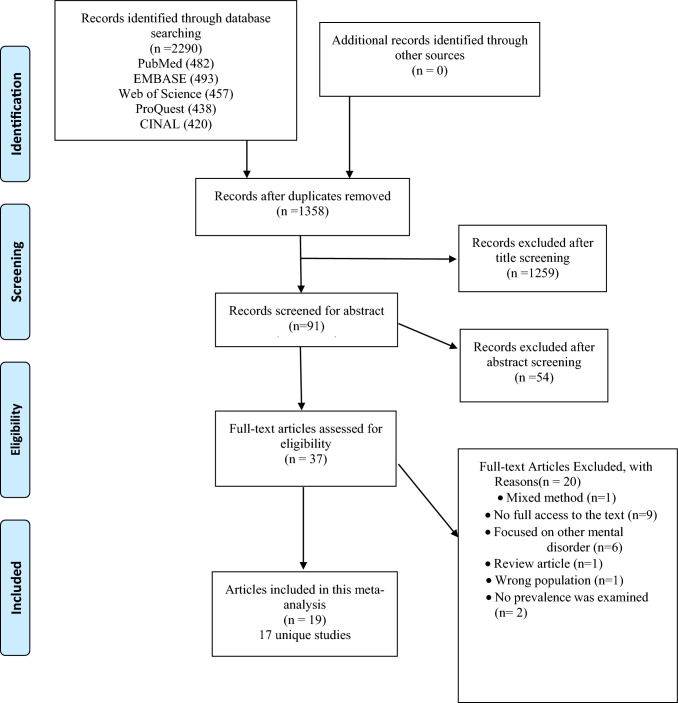
PRISMA diagram

Before we began our formal screening process, two reviewers (SE, Ch K) conducted a pilot screening exercise to refine our screening process and to test our data extraction sheet so that biases could be minimized. Selection of articles for inclusion involved a number of distinct steps. First, two reviewers (SE, Ch K) independently screened the titles and the abstracts of all eligible articles to determine whether they met the inclusion criteria using the data extraction sheet that had successfully been piloted for its utility. The same two reviewers then independently assessed the full text of the selected studies, resolving any disagreements between them through discussion until they achieved consensus. SE, as the primary author, then prepared two lists: (1) the eligible articles to aid her with the data extraction (discussed previously), and (2) the non-eligible articles with our reason(s) for excluding them. The PRISMA flow chart in Fig. 1 illustrates our study selection processes and the reasons for an article’s exclusion.

### Data Items

SE extracted data from each article about the characteristics of the study, the participants, and the outcomes. Data on **study characteristics** included study identification items (first author, year of publication, funding source), study design (cross-sectional, case–control, cohort), study setting (rural/urban), sampling method (random, non-random), sample population size, period of data collection, response rate, and the type of data collection (prospective or retrospective), Data on **participants’ characteristics** comprised inclusion criteria, recruitment setting (clinic, community), recruitment method (advertisement, phone contact), age (mean, range), gender (counts, proportions), education level, marital status, country of origin, working status (employed, unemployed), type of immigration into the U.S. (immigrant, refugee), and length of residency. Data on **outcome characteristics** included the outcome assessed (depression, anxiety), duration of mental health symptoms, method used to document mental health symptoms (validated or non-validated self-report instrument, clinical interview), outcome measurement instrument, type of reported statistics (counts, percentages, mean (standard deviation [SD]), odds ratio, risk ratio), and time point when the outcome was assessed.

### Outcomes and Prioritization

In light of the significant morbidity associated with depression and the clinical attention provided it [10, 25], our primary outcome of interest **originally** was depression. Depression is defined in the Diagnostic and Statistical Manual of Mental Disorders as “a persistent sadness and lack of interest associated with an inability to carry out daily activities” [26]. Depressive symptoms may range in intensity from mild to moderate and include some of the following: anxiety; sad mood; anergia**;** anhedonia; change in appetite; change in sleeping pattern; restlessness feelings of either worthlessness, guilt, or hopelessness or suicidal thoughts [27]. However, anxiety is not only a symptom of depression but a distinct mental disorder which often occurs concomitantly with depression [28, 29]. Anxiety may present with symptoms such as restlessness or feeling keyed up or on edge, irritability, muscle tension, and difficulty concentrating as well as sleep disturbance and fatigue [30]. In the various studies, depression and anxiety were defined either by clinical diagnosis, via a structured clinical interview, by a self-report measure, or through medical records.

### Risk of Bias in Individual Studies

Using a checklist adopted by Hölzel et al. [31], two reviewers (SE, Ch K evaluated the risk of bias in the articles, along with the articles’ research quality. The evaluation instrument followed the Critical Appraisal Skills Program (CASP) approach suggested by the British National Health Service and other research standards [30, 31]. The checklist is widely utilized to evaluate the internal and external validity of observational studies [31]. It comprises criteria for determining potential bias in six categories: selection, measurement, attrition, loss, whether the study has the external validity of randomized selection, and whether the sample was representative. The responses to the checklist questions pertaining to the six criteria consist of “Yes”, “No,” or “Unclear”; only longitudinal or cohort studies could satisfy the criteria for attrition bias or loss bias. Rather than just take the sum of the six scores, we conducted a more comprehensive evaluation of the methodological quality following the method specified by Hölzel et al. [31]. We applied only the four criteria pertinent to cross-sectional studies and case–control studies when evaluating them, yielding a maximum score of 4. Studies that fulfilled three or more of the instrument’s criteria were considered “high quality”, those satisfying 2 of the criteria as “medium quality” and those meeting less than 2 criteria as “low quality”. For cohort studies, the possible total score was 6. Those fulfilling four or more instrument quality criteria (out of six) were classed as “high quality”, those satisfying three criteria as “medium quality, and those meeting fewer than three criteria as “low quality”.

### Data Synthesis

Following the Cochrane recommendations [32], we constructed a systematic narrative synthesis using the information contained in each published source study’s text and tables to summarize and explain that study’s characteristics and findings. We listed, tabulated, and qualitatively reported the characteristics of our reviewed studies, including the participants, outcome measure (s), and the study quality (risk of bias). We subcategorized the studies based on the type of study design, publication year, and sample population size. We stratified our synthesis of the patient population (including mean age, age range, gender, and country of origin) by their immigration status (i.e., immigrants versus refugees). We summarized outcome characteristics, categorizing outcomes as either primary (depression) or secondary (anxiety), along with the name of the assessment instrument employed. Lastly, we described the quality of our reviewed studies in a table stratified by the overall methodological quality (low, moderate, or high quality).

### Meta-analysis

To enhance the precision of our systematic review and testing of our aims, we performed a meta-analysis of study results. Our analyses were conducted using the STATA software version 16.1. Based on our assumption that the studies in our review constituted a random sample and due to the expected heterogeneity across the studies, we used a random effects model to estimate a pooled prevalence of depression or anxiety, along with the associated 95% confidence intervals [33]. We measured the studies’ heterogeneity using Cochran’s Q chi-square and the I^2^ value [22]. The I^2^ statistic is a measure of the percentage of variation between our reviewed studies’ heterogeneity compared with the heterogeneity due to chance. It is categorized as follows: I^2^ < 25% indicates a low level of heterogeneity, between 25 and 50% indicates a medium level of heterogeneity, and > 50% indicates a high level of heterogeneity [33]. Cochran’s Q test enabled us to determine whether there were significant differences between prevalence estimates; a *p*-value of less than 0.05 is typically used to indicate that estimates are heterogeneous and do not follow the same distribution.

We used the subgroup analysis method appropriate for categorically defined subgroups of studies to examine our second aim and possible sources of heterogeneity between studies, The pooled effects of specific moderators were of interest: gender composition (female only, mixed male and female; there was no studies explored male only), immigration status (immigrants (those who migrated with their will, refugees (those who forced to leave their country), ethnicity (Syrian, Iraqi, mixed ethnicity (any other ethnic groups). This mixed category was reported in the included studies and specified to capture the diversity of ethnic backgrounds beyond singular Syrian or Iraqi origins. We also assessed effects of specific study characteristics, including study design type (prospective, retrospective, cross-sectional) and study research quality (high, moderate, low).

Thirdly, we performed a random effects meta-regression to investigate the impact of additional factors that may have influenced the effect sizes across studies. Based on the findings of previous reviews [21–23] combined with our clinical expertise, we chose to assess the following six characteristics of each study of its population as potential effect modifiers: total sample population size, publication year, the mean age of the Arab participants, proportion of the total number of participants who were female, the proportion of participants who were married, and the proportion of Arabs who had achieved less education.

Finally, we used two methods to examine our potential publication bias: (1) a visual inspection of our funnel plot [34–36] of the primary outcome measures, and (2) the performance of Egger’s intercept test to quantify the bias we might have seen in the funnel plot as well as to test whether the bias was statistically significant. We set our alpha criterion for Egger’s test to 0.05.

## Results

Our database search yielded a total of 2, 290 abstracts from a combination of PubMed (482 abstracts), ProQuest (438), Embase (493), Web of Science (457), or CINAHL (420). The process by which we winnowed through the abstracts to achieve our final set of review-eligible articles, together with the reasons we excluded the majority of articles, is summarized in our PRISMA diagram (Fig. 1). After we removed all duplicate entries, a total of 1358 potentially relevant unique articles remained. After we screened each article’s title, a total of 91 pertinent articles were eligible for abstract evaluation. After our review of the articles’ abstracts, 37 papers remained eligible for full-text analysis. After we had completed the full-text analysis, 19 publications (representing 17 distinct studies) passed all our screening criteria.

### Characteristics of Reviewed Studies and Study Participants

Our review includes 17 distinctive research projects, with a total population of 22, 558 participants, published between 2002 and 2022 (Table 2). Individual sample population sizes ranged from *N *= 25 to *N *= 18, 072. Of these studies, 13 were cross sectional, 2 were retrospective, and 2 were pilot studies. Eleven recruited their sample populations using a community-based approach (i.e., they recruited specifically from different Arab community organizations), from the researchers’ social networks or by word of mouth, or from Arab refugee centers. Two recruited participants from health clinics; more specifically, one of these enrolled subjects from primary care clinics in the Detroit area and one enrolled its participants from a mental health clinic in Michigan [37]. Three obtained their data by reviewing the participant’s electronic medical chart or health records [17, 18, 38–40].

**Table 2:** Study and participants characteristics

Author, Year	Study design	Sample size	Sample recruitment source	Immigration status	Ethnic group	Age (years)	Gender	Mental health symptoms	Outcome measurement
Abuelezem et al. (2022) and Abuelezem et al. (2021)	Cross- sectional	18, 072 Arab Americans and 5777 Iranian Americans	Electronic health records (EHRs) from a 2016 Northern California health plan cohort	Arab American	Ethnicities of Arab Americans from 22 countries, Iranian Americans	Range: 35–84 Mean age for Arab Americans of both genders was 532	M:507% F:493%	Depression or Anxiety	Clinical diagnosis of anxiety or depression
Abuelezam and El-Sayed (2021)	Cross- sectional	354 Arab American and 3517 non-Arab/ non—Hispanic whites	Arab Community Center for Economic and Social Services (ACCESS) from the standalone 2013 Michigan Arab Behavioral Risk Factor Survey (MI ABRFS)	Arab American	NR	Range: 18–40 +	M:534% F:466%	Depression	Reported health professional depression diagnosis
Suleiman and Whitfield (2021)	Cross- sectional	142	Community (Arab Community Center for Economic and Social Services (ACCESS) facility in Dearborn, Michigan ACCESS	75% Immigrants	Mixed Arab ethnicity ( Iraqi (35%), Lebanese (13%), Syrian (13%), Yemenite (13%), US-born (15%))	Range: 18 +	M:30% W:70%	Depression	CESD
M’zah, Cardozo and Evans (2019)	Cross- sectional	25	Community organization supporting Syrian refugees in Metropolitan Atlanta	Refugees	Syrian	Range: 18 + Mean age 375 (94)	M: 376% F: 373%	Anxiety symptoms, and depression	HSC 25 used for anxiety, depression
Javanbakht et al. (2019)	Cross- sectional	157	Two primary care clinics in the Detroit area of Michigan	Refugees	Syrian	Range: 18–65 Mean age: 3608 (1141)	M: 529% F:471%	Anxiety and depression	HSC-25
Javanbakht et al. (2018)	Cross- sectional	131	Children from two primary care clinics in the Detroit area of Michigan	Refugees’ children	Syrian	Range: 6–17 years Mean age 1102 (332)	M: 595% F: 405%	Anxiety and depression	The SCARED instrument for anxiety, HSC-25 for depression
Aroian, Uddin and Pham (2015)	Cross- sectional	538	Researcher community network	Arab immigrants (447%) refugees (445%)	Mixed ethnicity—Iraq (437%), Lebanese (338%)	Range: 18 + Mean age 4024 (647)	Female only	Depression	CESD
Jaber et al. (2015)	Cross- sectional	98	The ACCESS Child and Adolescent Health Center and the Helping Youth Progress and Excel (HYPE) Recreation Center, Dearborn, Michigan	Arab immigrants (- generation immigrants (69%), first- generation (20%), third generation (10%))	Mixed ethnicity (mostly US born (79%), Saudi (5%), Yemen(5%), Lebanon(5%), Iraq(4%))	Range: 12–17 Mean age 154 (15)	M:57% F:43%	Depression	PHQ9
Kira et al. (2014)	Cross- sectional	399	Mental health clinic	Arab Americans (688%) and refugees (312%)	Mixed (Iraqi, (278%), Lebanese (278%), Yemenis (215%), other Arabs (56%)	Range: 18–76 Mean Age 66 (1145)	M:535% F:464%	Depression or anxiety	CESD, DASS for anxiety and depression
Tylor et al. (2014)	Cross- sectional	366	Local refugee resettlement agencies’ lists	Refugees, or on special immigrant visas	Iraqi	Range: 18 +	M:60% F: 40%	Anxiety or depression	HSC-25 for anxiety, depression
Amer and Hovy (2012)	Cross- sectional	601	Email invitation to Arab American personal contacts	Arab American	Mixed Arab ethnicity (Palestinian (245%), Egyptian (198%), Lebanese(161%), Syrian (68%), Iraqi(5%))	Range: 18–81 Mean age 293 (111)	M:38% F:62%	Depression, anxiety	CESD for depression; BAI for anxiety
Grant& Keltner, (2011), Norris et al., (2011)	Cross- sectional	519	Arab immigrant women living in metropolitan Detroit	Immigrants	Mixed Arabs (Iraqi (541, Lebanese (331%), born in the Middle East or Northern Africa(128%)	Mean age 4022 (65)	Female only	Depression	CESD
Jamil et al. (2010)	Pilot	285 (Iraqi refugees (n = 191), non-refugees (n = 94))	Psychiatric medical chart review	Refugees, non- refugees	Iraqi	Range: 18 +	M: 465% F:355%	Major depression	Clinical assessment by psychiatrist in medical chart
Abu-Ras and Abou badr (2009)	Cross- sectional	350	Community-based, health care, and religious organizations in California, Michigan, New York, New Jersey, and Washington, DC	Arab American	Mixed Arab ethnicities (22 countries), including Egyptian, Bahraini, Syrian, Tunisian, Algerian)	Range: 18–77 Mean age 344 (1171)	M: 45% F: 55%	Depression	CESD
Hassouneh and Kulwicki (2007)	Pilot	30	Personal networks, informational flyers	Immigrants	Palestine (334%), Iraqi (30%), Lebanese (233%), Syrian (67), Yemeni(33%) and African American (33%)	Range: 27–65 Mean age 38 (1042)	Female only	Depression, anxiety	BDI-II and CESD used for depression; BAI for anxiety
Jamil (2007)	Retrospective	116	Refugees’ health center medical records	Refugees	Iraqi	Range: 19–75 Mean age 518 (317)	M:604% F:396%	Depression, anxiety symptoms	HSC-25
Jamil et al. (2002)	Retrospective	375	Review medical charts of clients who sought mental health services at the Arab Community Center for Economic and Social Services (ACCESS) in Dearborn, Michigan, from 1998 to 1999	Refugees	Iraqi	18–40 +	M: 621% F: 379%	Depressive disorders	Medical diagnosis by mental health professionals

NR (Not reported), F(Female), M(Male), NHIS (National Health Interview Survey). Depression measures: CES-D (Center for Epidemiological Studies Depression Scale), CESD-SF (Center for Epidemiologic Studies Depression—Short Form), BDI-II (Beck Depression Inventory II), GDS (Geriatric Depression Scale), PHQ9(patient Health Questionnaire-9), PHQ4 (Patient Health Questionnaire-4), HSC-25 (Hopkins Symptom Checklist 25 items). Anxiety measures: SCARED (The Screen for Child Anxiety Relied Disorders), GAD-4(Generalized Anxiety Disorders), DASS (Depression Anxiety Stress Scales), BAI (Beck Anxiety Inventory)

Regarding their participants’ ages, two studies [8, 37, 40, 41] recruited children or adolescents, ages 6 to 17 years, while fifteen studies enrolled Arab adults (> = 18 years of age) in mixed age groups. Although 14 studies reported data on Arab populations of both genders, three were focused exclusively on Arab women. The participants’ immigration status varied, with some of our reviewed studies including U.S. born Arabs. Five of the studies focused solely on Arab immigrants of multiple generations, and seven considered only Arab refugees. Five studies recruited from the Arab American population, either U.S.-born or naturalized citizen, and six recruited populations of mixed immigration status, i.e., U.S.-born Arab Americans, immigrants, refugees, asylum seekers, tourists, or special immigration visa holders. Nine studies (slightly more than half of studies) had recruited Arabs of mixed ethnicity; the most reported countries were Syria, Lebanon, Iraq, Palestine, and Yemen. Four enrolled only Iraqi Arabs, three examined Syrian Arabs, and one did not provide information regarding the ethnicity of the study populations.

### Clinical Outcomes and Measurements

Clinical outcomes were classified as either depression or anxiety. All reviewed studies (*n *= 17) evaluated participants ‘depression level. Only eight reported investigating depression as the sole outcome; of these, Jamil et al. [18] focused on major depression. The most frequently reported depression assessment instrument used (six studies) was the Center for Epidemiological Studies Depression Scale (CES-D), either the 10-item version or the 20-item version [9, 37, 42–47]. Five studies indicated the use of the 25-item Hopkins Symptom Checklist, or HSC-25 [17, 25, 48–50]. For four studies, a depression diagnosis was reported to have been established through professional clinical assessment rather than via self-report [18, 38–40]. One indicated the use of the Beck Depression Inventory-II (BDI-II, 21items), and one reported employing the Patient Health Questionnaire-9 [41, 51].

Nine studies reported evaluating anxiety concurrently with depression. No studies have exclusively evaluated the participants anxiety. Anxiety symptoms of differing degrees of specificity were reported as being assessed using different instruments. Five studies used the previously mentioned HSC-25 measure [17, 25, 48–50]. Two reported examining generalized anxiety disorder with the Beck Anxiety Inventory [44, 51], and Kira et al. used the Depression Anxiety and Stress Scale [37]. Javanbakht et al. [25] stated that they had used the Screen for Child Anxiety Related Disorders.

### Prevalence and Severity of Depression and Anxiety

**Fig. 2:**
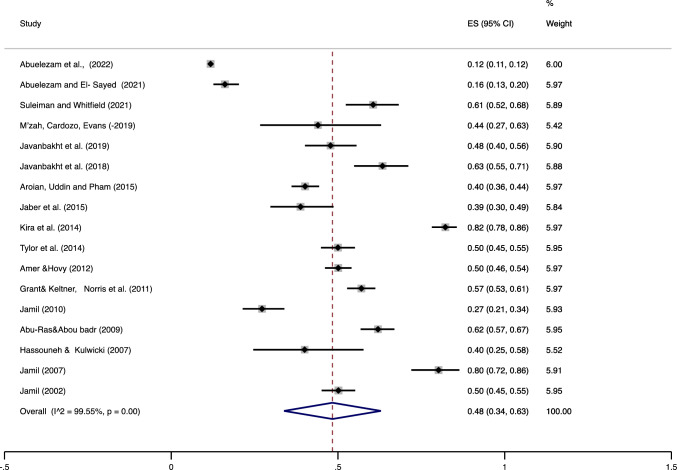
Pooled prevalence of depression among Arab immigrants

Seventeen studies (22, 558 participants) were included in estimating the prevalence of depression for Arabs living in the U.S. (Fig. 1); our estimate of the pooled prevalence of depression among these studies was 48%, 95% CI ((34%, 63%), Q value = 3583.50 (df = 16)). The I^2^ value (I^2^ = 99.55, *p* < 0.001) indicated the existence of a large amount of heterogeneity in the reported prevalence of depression (99.55%). Data from 10 studies (*N *= 19, 978) was used to obtain a similar estimate for anxiety (Fig. 2); our estimate of the pooled prevalence for anxiety was 58%, 95% CI ((33%, 83%), Q value = 2498.56 (df = 8)). Similar to our depression prevalence estimate, we found a significantly large amount of heterogeneity (99.68%) across studies (I^2^ = 99.57, *p* < 0.001).

### Sub-Group Analysis for Moderation

We summarized our estimates of the pooled prevalence for depression and anxiety by subgroup in Table 3. In terms of gender, depression was more prevalent in mixed-gender studies (49%, 95% CI [32%, 66%]) than in the female-only studies (47%, 95% CI [33%, 60%]). Similarly, anxiety was more prevalent for the mixed-gender studies (97%, 95% CI [83%, 99%]) than for the women-only studies (53%, 95% CI [29%, 77%]). Regarding participants’ immigration status, depression was most prevalent in studies which combined Arab individuals with a variety of immigration statuses in their samples (e.g., U.S. born, refugees, visa holders) (71%, 95% CI [68%, 74%]), followed by those Arab participants who were strictly members of refugee groups (52%, 95% CI [37%, 68%]), and Arab immigrants had the least depression. The largest prevalence of anxiety also was observed among Arab individuals of mixed immigration status (64%, 95% CI [61%, 67%]), followed by refugees (61%, 95% CI [43%, 81%]), and immigrants, who experienced the lowest rate (12%, 95% CI [11%, 12%]). In terms of ethnicity, those participants who were either Iraqis or Syrians had the highest rates of depression (52%, 95% CI [33%, 77%]) compared to Arabs of mixed ethnicity, who showed a depression rate of 46% (95% CI [28%, 64%]). Mixed ethnic Arabs (i.e., with heritage from a variety of countries) had a slightly larger rate of anxiety (57% (95% CI [17%, 97%]) compared to those of either Iraqi or Syrian origin (56% (95% CI [46%, 68%]).

**Table 3:** Subgroup analyses of the prevalence of depression and anxiety among Arab individuals

Subgroup Comparison	Pooled prevalence of depression				Pooled prevalence of anxiety			
	# of Studies	Pooled Prevalence	95% CI		# of Studies	Pooled Prevalence	95% CI	
			Lower limit	Upper limit			Lower limit	Upper limit
*Gender composition*								
Women only	3	0.47	033	0.60	1	0.53	0.29	0.77
Mixed	14	0.49	0.32	0.66	9	0.97	0.83	0.99
Total	17	0.48	0.34	0.63	10	0.58	0.33	0.83
*Immigration status*								
Refugees	6	0.52	0.37	0.68	4	0.61	0.42	0.81
Immigrants	4	0.42	0.11	0.74	2	0.12	0.11	0.12
Arab American	5	0.41	0.25	0.58	1	0.47	0.43	0.51
Mixed	2	0.71	0.68	0.74	3	0.64	0.61	0.67
Total	17	0.48	0.34	0.63	10	0.58	0.33	0.83
*Ethnicity*								
Iraqi or Syrian	5	0.52	0.33	0.72	4	0.56	0.46	0.68
Mixed	12	0.46	0.28	0.64	6	0.57	0.17	0.97
Total	17	0.48	0.34	0.63	10	0.58	0.33	0.83
*Study type*								
Cross-sectional	13	0.48	0.31	0.65	8	0.50	0.25	0.74
Pilot	2	0.29	0.33	0.35	1	0.97	0.83	0.99
Retrospective	2	0.60	0.56	0.64	1	0.80	0.72	0.86
Total	17	0.48	0.34	0.63	10	0.58	0.33	0.83
*Study quality*								
High	4	0.32	0.11	0.52	3	0.36	0.07	0.66
Medium	7	0.57	0.40	0.75	6	0.65	0.49	0.81
Low	6	0.51	0.44	0.58	1	0.93	0.87	0.99
Total	17	0.48	0.34	0.63	10	0.58	0.33	0.83

*df* degree of freedom

Differences in the estimated prevalence of depression were also found among studies using varied types of research design and studies having varied research quality. Retrospective studies reported the largest rate of depression (60%, 95% CI [56%, 64%]), with cross-sectional studies reporting the second largest rate (48%, 95% CI [31%, 65%]), and pilot studies the lowest rate (29%, 95% CI [33%, 35%]). The prevalence of anxiety was the largest for the pilot or the retrospective research types (97%, 95% CI [83%, 99%] and 80%, 95% CI [72%, 86%], respectively) compared to the cross-sectional study designs (50%, 95% CI [25%, 74%]). When comparing studies of different research quality, the medium-quality and the low-quality studies reported a larger prevalence of depression than the high-quality studies (57%, 95% CI [40%, 75%]; 51%, 95% CI [44%, 58%]; and 32%, 95% CI [11%, 52%], respectively). Similarly, studies of poor quality reported a larger anxiety prevalence (93%, 95% CI [87%–99%]) compared to the medium-quality studies (65%, 95% CI [49%, 81%]) or the high-quality studies (36%, 95% CI [7%, 66%]).

### Meta Regression of Additional Factors Influencing Prevalence Results

Table 4 includes meta-regression results for their effect of other variables on the prevalence of depression and anxiety across studies.

**Table 4:** Results of the random effects meta-regression of the studies and demographic moderators for the prevalence depression and anxiety

Moderator	Depression					Anxiety				
	Coefficient	Standard error	t-value	p-value	95% CI	Coefficient	Standard error	t-value	p-value	95% CI
Total Sample Size	− 0.0025	0.00012	− 1.83	0.087	0.0005, 3.71	− 0.00024	0.00014	− 1.76	0.117	− 0.00056, 0.0044
Publication Year	− 0.022	0.0089	− 2.55	*0.02	− 0.040, − 0.004	− 0.0146	0.0167	0.087	0.410	− 0.529, 0.0239
Mean age of Arab Individuals	− 0.0085	0.0054	− 1.55	0.152	− 0.0206, 0.0036	− 0.0118	0.0028	− 4.10	**0.005	− 0.0186, − 0.0050
Proportion of Female Arabs	− 0.00026	0.0033	0.78	0.453	− 0.0048, 0.010039	0.1295	0.3517	0.37	0.728	− 0.775, 1.034
Proportion of Married Arabs	− 0.0627	0.4461	− 0.14	0.892	− 0.0156, 0.9660	− 0.597	0.4054	− 0.15	0.890	− 1.185, 1.065
Proportion of Arab with lower education (high school or less)	− 0.0073	0.0051	− 1.43	0.191	− 0.0189, 0.0045	0.0258	0.4552	0.06	0.958	− 1.423, 1.475
Ethnicity Being Iraqi or Syrian	0.232	0.1265	1.84	0.086	− 0.0369, 0.5021	0.209	0.1614	1.30	0.230	− 0.162, 0.582

*CI* Confidence Interval.

#### Depression

Only one variable we examined was associated with the prevalence of depression. Publication year was negatively related to the prevalence of depression, with older publications being more likely to report higher prevalence rates (β = − 0.022, *p* = 0.02, 95% CI − 0.040, − 0.004). Total sample population size in publications was not associated with the prevalence of depression (β = − 0.0025, *p* = 0.087, CI 0.0005, 0.00371). Mean age of Arab participant was not associated with depression prevalence (β = − 0.0085, *p* = 0.152, 95% CI − 0.0206, 0.0036). The proportion of female Arab participants did not appear to influence the effect size of depression prevalence (β − 0.00026, *p* = 0.453, 95% CI − 0.0048, 0.01003). Similarly, there was no relationship between depression prevalence and either the proportion of married participants or those who had achieved less education (β = − 0.063, *p* = 0.892, 95% CI − 0.0156, 0.0966) and (β = − 0.0073, *p* = 0.191, 95% CI − 0.0189, 0.0045), respectively.

#### Anxiety

Participants’ mean age was negatively related to reported prevalence of anxiety in publications, indicating diminishing prevalence of anxiety among individuals as their age increased (β = − 0.0118, *p* = 0.005, 95% CI − 0.0186, − 0.0050). None of the other variables we examined were related to the effect size of anxiety prevalence, including year of the publication (β = − 0.0146, *p* = 0.410, 95% CI − 0.529, 0.0239), sample size (β = − 0.0024, *p* = 0. 117, 95% CI − 0.0056, 0.0044), proportion of married Arab participants (β = − 0.062, *p* = 0.892, 95% CI − 0.0156, 0.966) or the proportion of Arabs who completed less education (β = − 0.0073, *p* = 0.086, 95% CI − 0.036, 0.502).

### Methodological Quality of the Studies

**Fig. 3:**
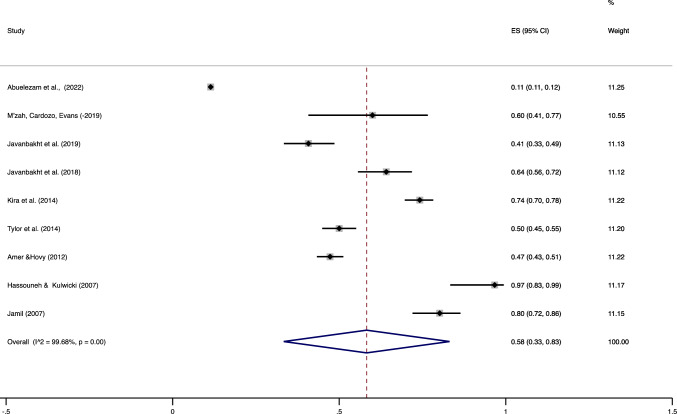
Pooled prevalence of anxiety among Arab immigrants

About four (23%) of our reviewed studies were classified as being of greater quality, at least in terms of their methodology (Tables 5). Of these, only one (5.7%) met all four of the criteria for cross-sectional studies [50]. Four (23%) satisfied three of the same criteria [8, 38, 39, 44, 50]. Nearly half of the studies (42%, (*n *= 7)) were of “medium quality”, and 6 (35%) studies were identified as “low quality”. None of the studies we reviewed violated all of the criteria we used to assess their methodological quality, meeting at least one criterion (Fig. 3).

**Table 5:** Methodological quality of the included studies

Author (year)	1. Selection Bias: Comparable Study Groups or Statistical Adjustment	2. Measurement Bias: Valid and Reliable Measurement of the Exposure	5. External Validity: Consecutive or Randomized Selection	6. External Validity: Representative Sample	Overall Evaluation of Methodological Quality
Abuelezem et al. (2022), Abuelezem et al. (2021)	** + **	** + **	**–**	** + **	High
Abuelezam and El- Sayed (2021)	**0**	** + **	** + **	** + **	High
Suleiman and Whitfield (2021)	** + **	** + **	**–**	**–**	Medium
M’zah et al. (2019)	**0**	** + **	**–**	**–**	Low
Javanbakht et al. (2019)	** + **	** + **	**–**	**–**	Medium
Javanbakht et al. (2018)	** + **	** + **	**–**	**–**	Medium
Aroian et al. (2015)	**0**	** + **	**0**	** + **	Medium
Jaber et al. (2015)	**0**	** + **	**–**	**0**	Low
Kira et al. (2014)	** + **	** + **	**–**	**–**	Medium
Tylor et al. (2014)	** + **	** + **	** + **	** + **	High
Amer and Hovy (2012)	** + **	** + **	**–**	** + **	High
Grant and Keltner (2011), Norris et al. (2011)	**0**	** + **	**–**	**0**	Low
Jamil (2010)	** + **	** + **	**–**	**–**	Medium
Abu-Ras and Abou badr (2009)	**–**	** + **	**–**	** + **	Medium
Hassouneh and Kulwicki (2007)	**0**	** + **	**–**	**–**	Low
Jamil, 2007	**–**	** + **	**–**	**–**	Low
Jamil, 2002	**–**	** + **	**–**	**–**	Low

(*) Codes used are as follows: +  = criterion met, – = criterion not met, 0 = missing information or unclear criterion.

### Publication Bias

**Fig. 4:**
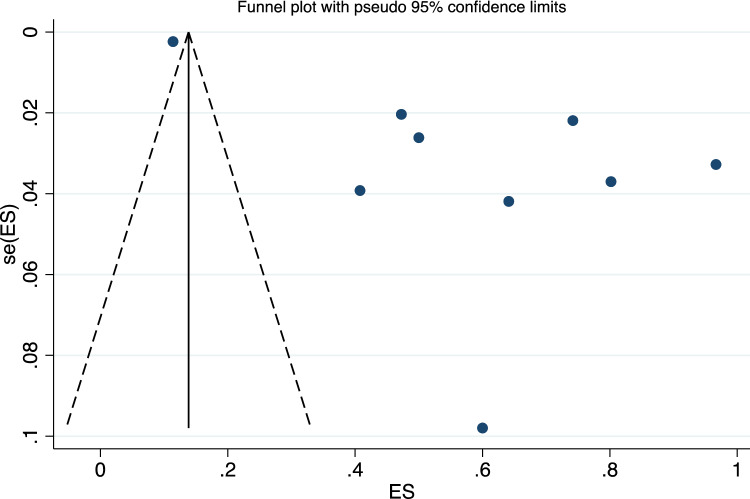
Funnel plot for the studies’ publication bias

As indicated by the quite symmetric nature of the plotted study size versus the plotted effect size in the funnel plot (Fig. 4, there is little evidence of publication bias; in the following text, we use the term “the left side” to refer to the area of the funnel plot in which the effect size is 0 (the natural log of 1), or less; similarly, we use the phrase the “right side” to describe those effect sizes greater than 0. Figure 4 shows set of studies which appear on the left side of the funnel plot are more dispersed, i.e., these studies may not have reported assessments of effect size close to the true effect size or may have recruited sample populations varying more in size, and one study appearing on the right side of the plot (which more likely reported effect sizes close to the true effect size or tended to recruit sample populations closer to each other in size). Our Egger’s Test results (*p* = 0.138), with alpha set to 0.05, provide no significant evidence for the presence of publication bias. This suggests the accuracy and reliability of the pooled estimates we found.

## Discussion

The number of displaced or immigrant Arabs living in the U.S. has increased greatly; the sociocultural challenges associated with immigration have increased concomitantly their risks of suffering debilitating mental health problems such as depression and anxiety [2, 12]. However, there has been no synthesis of existing knowledge to date regarding the actual prevalence of depression and anxiety in this vulnerable population. In this meta-analysis, we have addressed this knowledge gap to some extent.

### Prevalence of Anxiety and Depression

Although our analyses indicated significant heterogeneity across studies in their results, our pooled estimates suggest high prevalence rates of 48% for depression and 58% for anxiety among Arab individuals in the U.S. These results are very troubling and underscore the urgent need to address mental health concerns in Arab communities, given the potential impact on individuals’ well-being and social functioning [52]. Previous researchers have suggested that immigrant groups are indeed more likely to exhibit symptoms or to develop a mental disorder than their counterparts remaining in their country of origin [20]. Our results suggest that Arab refugees suffer more from depression r anxiety than Arab immigrants do. Turrini et al., in their umbrella review of refugees worldwide, reported that depression, anxiety or PTSD sufferers accounted for up to 40% of asylum seekers and refugees [29]. Lindert et al. in their review concluded that worldwide refugees resettled in any country showed a greater prevalence of depression and anxiety than immigrants who come to a country seeking work and a better life [14]. This previous evidence, as well as our results, suggest that emigrants fleeing violence or religious oppression in their countries of origin are more traumatized and have a worse mental health status than those who leave their home country deliberately in search of professional or economic mobility [7, 14]. However, the pronounced heterogeneity we found among our studies signifies potential differences in cultural perceptions of depression or anxiety, differences in the methodological or diagnostic criteria employed in assessing these mental health problems, or the socio-economic context in which various studies were conducted [52, 53].

### Subgroup Analysis for Moderating Effects

In contrast to women-only studies, research using mixed samples of men and women in this review reported a greater prevalence of both depression and anxiety. These findings contradict some results reported in the literature, suggesting that women are more vulnerable to mood disorders and depression [54, 55]. However, some of the studies we reviewed reported no significant gender differences [56]. Ultimately, it is the intersection of societal and cultural factors with gender that may produce varying results across different contexts and studies.

In our analyses, we also found varying prevalence rates of depression or anxiety among different immigrant groups, with studies that integrated multiple types of Arab immigrants having the highest prevalence rates. It is likely that these studies achieved the most robust rates by capturing many facets of the complex acculturation process, identity conflicts, and challenges associated with integrating into a new cultural context [57]. In addition, the larger prevalence rates for depression demonstrated by Arab refugees in this review are consistent with a considerable number of reports from the literature describing the psychological effects of forced displacement as well as their exposure to traumatic events [21, 58, 59]. The fact that other categories of Arab immigrants had lower rates than refugees may reflect protective factors associated with successful integration, social support, and selective migration that are more characteristic of these non-refugee groups [52, 60].

We found considerable variation in the rates of depression and anxiety by study design type. For the cross-sectional studies, which dominated our analysis, we identified a moderate prevalence of depression and anxiety. Due to the limited temporality of this design type, it cannot indicate changes over time. By contrast, retrospective studies reported larger estimates for depression or anxiety, which could stem from recall bias, and other methodological issues [61]. However, it might also reflect a more comprehensive, historical assessment of the individual’s mood state which is not restricted by one point in time.

It is particularly important to note that the highest quality studies within our review (in terms of research rigor and reduced bias) reported the lowest prevalence rates for both depression and anxiety. This likely signifies more accurate assessments of prevalence by these studies as a result of their enhanced controls for error variance associated with sample selection and retention, measurement, and other features of their study designs.

### Other Factors Influencing Prevalence Rates

Our meta-regression analysis highlighted the role of publication year and age of the individual as factors influencing the prevalence of depression and anxiety, respectively. The significant inverse relationship we observed between publication year and depression prevalence suggests a potential decreasing trend in Arabs’ depression rates. This view aligns with some previous research that suggests Arabs may enjoy an improved societal awareness, reduced stigma, and increased access to mental health services than in previous years [57]. However, some research indicates a stable prevalence of depression or a trend towards an increase in depression prevalence [62]. The complexity of the evolving patterns of depression or anxiety underscores the need for ongoing surveillance and rigorous exploration of these dynamic trends.

The significant negative relationship we found between Arab participants’ mean age and the prevalence of anxiety seemingly comply with the work of Craske et al., which suggested a decrease in anxiety with increasing age. Hence, although age is a significant risk factor for anxiety, it should be considered within a broader framework that encompasses other determinants of an Arab individual’s anxiety [63]. Future researchers should explore the interplay of age with other contextual factors and should conduct longitudinal studies to examine more comprehensively the evolution of anxiety across the lifespan within Arabic communities.

The non-significant associations we observed between other demographic factors and prevalence rates suggests they may not be direct drivers of Arabs’ depression or anxiety. This agrees with previous work indicating complex interactions between these factors and mental health outcomes [7, 11].

### Limitations

We recommend interpreting our review results with some caution due to varied study limitations First, restricting our search to studies published in English eliminated potentially eligible articles in other languages; this limits the generalizability of our findings. Second, the greater heterogeneity of our source studies and their publication bias may reduce the quality of our evidence, potentially altering the size of our estimates for prevalence as well as the strength of the associations we found for our subgroup analyses and meta-regressions. Additionally, the use of self-reported measures and variations in diagnostic criteria in our reviewed studies could have contributed to inconsistencies in prevalence rates. However, to the best of our knowledge, ours is the first meta-analysis to determine the pooled prevalence of depression and anxiety among Arab populations in the U.S. We also used an established methodology (the PRISMA-P guidelines) along with a comprehensive search strategy to identify a large number of relevant articles. Our assessment of potentially conflicting evidence through subgroup and meta-regression analyses exemplifies our thoroughness. By dissecting variations in prevalence based on factors such as gender, immigration status, and research quality, we present a nuanced portrayal of depression and anxiety among Arab populations in the U.S.

### Future Research Implications

The dearth we found in high-quality research regarding the prevalence of depression and anxiety among Arab immigrants living in the U.S. presents a significant barrier to providing appropriately informed mental health care for this population [25, 64]. Further understanding of Arab groups at particular risk for these mental health problems is essential so that tailored interventions can be developed to address depression and anxiety across different demographic subgroups, such as women, refugees and younger immigrants. Such interventions should consider cultural nuances, socio-economic factors, and access to healthcare services to meet more effectively address the diverse mental health needs of U.S. Arab residents. Additional research is also needed to explore in greater depth the causes of depression, anxiety, and other mental health problems among U.S. Arab population, both the first-generation immigrants as well as the second or later generations. Epidemiologic studies in the Arabic community ideally should include population-based sampling and diagnostic instruments validated in the appropriate language for the target community.

Our analyses suggest that upcoming research efforts should prioritize methodological rigor to improve the quality and reliability of findings. This includes using standardized methodologies, robust sampling techniques, increased efforts to reduce publication bias, and transparent reporting practices to ensure the validity of results. High-quality studies are essential for accurately assessing prevalence rates and identifying effective interventions for depression and anxiety. Longitudinal studies tracking mental health outcomes over time and comparative studies comparing mental health outcomes among U.S. Arab residents with those in their countries of origin or those observed in other immigrant populations will provide valuable insights into the impact of migration, acculturation, and socio-cultural factors on mental health. These studies can inform the development of targeted interventions and policies to address mental health disparities effectively. We also recommend that research be conducted in the middle-income or low-income host countries of Arab immigrants to compare the risk of mental disorders found in our review with those prevalent in those countries. Ultimately, our review and additional research may encourage clinicians to increase their awareness of and their commitment to screen for and treat mental health problems in their Arab patients.

## Conclusion

Our meta-analysis sheds light on the high prevalence rates for depression and anxiety experienced by resettled Arab populations in the U. S. Analysis of subgroup data and demographics suggested that factors such as gender, immigration status, country of origin, age, and research quality can significantly influence prevalence rates. Overall, findings underscore the imperative for tailored interventions and policy initiatives aimed at addressing the multifaceted mental health needs of resettled Arab communities in the U.S., and the urgent need to advocate for equitable access to culturally sensitive support and resources.

## Supplementary Information

Below is the link to the electronic supplementary material.

Supplementary file1 (DOCX 43 KB)
