# Correction: Depression and Anxiety among Arab Individuals in the United States: A Meta-analysis

**DOI:** 10.1007/s10903-024-01665-8

**Published:** 2025-01-20

**Authors:** Shaimaa Mosad El-Refaay, Christina Kenny, Sandra Weiss

**Affiliations:** 1 School of Nursing, UCSF, 2 Koret Way Rm 411Y, San Francisco, CA 94143 USA; 2 Division of Geriatrics, School of Nursing, UCSF, San Francisco, USA; 3 Department of Community Health Systems, Stress and Depression Lab, University of California, San Francisco, USA

**Correction to: Journal of Immigrant and Minority Health**

10.1007/s10903-024-01648-9

The original version of this article unfortunately contained errors in Tables 2, 3, 4, 5 and supplementary Tables 1 and 2.

The wrong Supplementary file has now been replaced with the correct file and the incorrect and corrected Tables 2, 3, 4 and 5 are given here.

**Incorrect version of Table 2**

**Table 2:** Study and participants characteristics

Author, Year	Study Design	Sample Size	Sample Recruitment Source	Immigration Status	Ethnic Group	Age (years)	Gender	Mental Health Symptoms	Outcome Measurement
**Patel et al. (2022)**	Cross-sectional	283	Community commercial, health care or religious settings in Michigan	NR	Mixed ethnicity (mostly Iraqi [369%] and Lebanese [33%])	Range: 18 and older	M:36% F:64%	Feeling depressed, anxious, nervous, or worried	PHQ4
**Javanbakht et al. (2022)**	Cross-sectional	152	Two primary care clinics serving most individuals resettling in Detroit, Michigan	Refugees	Syrian or Iraqi	Range: 18-65 Mean age 3633 (1154)	M: 507% F:493%	Depression and Anxiety	Hopkins Symptoms Checklist 25 (HSC-25) for depression, anxiety, and PTSD
**Abuelezem et al. (2022) and Abuelezem et al. (2021)**	Cross-sectional	18, 072 Arab Americans and 5777 Iranian Americans	Electronic health records (EHRs) from a 2016 Northern California health plan cohort	Arab American	Ethnicities of Arab Americans from 22 countries, Iranian Americans	Range: 35-84 Mean age for Arab Americans of both genders was 532	M:507% F:493%	Depression or Anxiety	Clinical diagnosis of anxiety or depression
**Abuelezam and El-Sayed (2021)**	Cross-sectional	354 Arab American and 3517 non-Arab/ non - Hispanic whites	Arab Community Center for Economic and Social Services (ACCESS) from the standalone 2013 Michigan Arab Behavioral Risk Factor Survey (MI ABRFS)	Arab American	NR	Range: 18-40+	M:534% F:466%	Depression	Reported health professional depression diagnosis
**Suleiman and Whitfield (2021)**	Cross-sectional	142	Community (Arab Community Center for Economic and Social Services (ACCESS) facility in Dearborn, Michigan ACCESS	75% Immigrants	Mixed Arab ethnicity (Iraqi (35%), Lebanese (13%), Syrian (13%), Yemenite (13%), US-born (15%))	Range: 18+	M:30% W:70%	Depression	CESD
**Arnetz et al. (2020)**	Cross-sectional	64	Refugees’ resettlement agency in Michigan	Refugees	Syrian or Iraqi	Mean age 3763 (118)	M:516% F: 484%	Depression and Anxiety	7-item depression subscale of the Hospital Anxiety and Depression Scale, Beck Anxiety Inventory for anxiety
**Kader et al. (2020)**	Cross-sectional	279	Arab Community Center for Economic and Social Services (ACCESS) in Dearborn, Michigan	Immigrants (56%), refugees (24%), US-born (20%)	Mixed ethnic groups	Range: 18–85	M: 39% F:61%	Depression or Anxiety	CESD for depression GAD for anxiety
**M’zah, Cardozo and Evans (2019)**	Cross-sectional	25	Community organization supporting Syrian refugees in Metropolitan Atlanta	Refugees	Syrian	Range: 18 + Mean age 375 (94)	M: 376% F: 373%	Anxiety symptoms, and depression	HSC 25 used for anxiety, depression
**Javanbakht et al. (2019)**	Cross-sectional	157	Two primary care clinics in the Detroit area of Michigan	Refugees	Syrian	Range: 18–65 Mean age: 3608 (1141)	M: 529% F:471%	Anxiety and depression	HSC-25
**Javanbakht et al. (2018)**	Cross-sectional	131	Children from two primary care clinics in the Detroit area of Michigan	Refugees’ children	Syrian	Range: 6–17 years Mean age 1102 (332)	M: 595% F: 405%	Anxiety	The SCARED instrument for anxiety
**Pampati et al. (2018)**	Cross-sectional	275	The Arab Community Center for Economic and Social Services (ACCESS) in Dearborn, Michigan	Immigrants (556%) refugees (244%), and US-born Arabs (20%)	NR	Range: 18–85 Mean age 387 (17)	M: 382% F: 618%	Depression or anxiety	CES-D-SF for depression, GAD-4 scale for anxiety
**Lemaster et al. (2018 )**	Longitudinal	298 at baseline, and 291 at 1-year visit	Community-based sample of newly arrived Iraqi refugees at seven local resettlement agencies	Refugees	Iraqi	Range: 18+ *Mean age at baseline, 334 (113) *Mean age at follow-up 343 (114)	NR	Depressive symptoms	Hospital Anxiety and Depression Scale for depression
**Arion, Uddin, and Blbas (2017)**	Longitudinal	388	Network sampling by 12 Arab immigrant research assistants in the local Metropolitan area of Detroit	Refugees (445%), immigrants (472%), student or tourist visa (82%)	Mixed Arab ethnicities - Iraqi (443%), Lebanese (365%), Yemenite (112%)	Range: 18 + Mean age 4297 (592)	Female only	Depression	CESD
**Aroian, Uddin and Pham (2015)**	Cross-sectional	538	Researcher community network	Arab immigrants (447%) refugees (445%)	Mixed ethnicity - Iraq (437%), Lebanese (338%)	Range: 18 + Mean age 4024 (647)	Female only	Depression	CESD
**Jaber et al. (2015)**	Cross-sectional	98	The ACCESS Child and Adolescent Health Center and the Helping Youth Progress and Excel (HYPE) Recreation Center, Dearborn, Michigan	Arab immigrants (-generation immigrants (69%), first-generation (20%), third generation (10%))	Mixed ethnicity (mostly US born (79%), Saudi (5%), Yemen (5%), Lebanon (5%), Iraq (4%))	Range: 12 to 17 Mean age 154 (15)	M:57% F:43%	Depression	PHQ9
**Kira et al. (2014)**	Cross-sectional	399	Mental health clinic	Arab Americans (688%) and refugees (312%)	Mixed (Iraqi, (278%), Lebanese (278%), Yemenis (215%), other Arabs (56%)	Range: 18 to 76 Mean Age 66 (1145)	M:535% F:464%	Depression or anxiety	CESD, DASS for anxiety and depression
**Tylor et al. (2014)**	Cross-sectional	366	Local refugee resettlement agencies’ lists	Refugees, or on special immigrant visas	Iraqi	Range: 18+	M:60% F: 40%	Anxiety or depression	HSC-25 for anxiety, depression
**Amer and Hovy (2012)**	Cross-sectional	601	Email invitation to Arab American personal contacts	Arab American	Mixed Arab ethnicity (Palestinian (245%), Egyptian (198%), Lebanese (161%), Syrian (68%), Iraqi (5%))	Range: 18–81 Mean age 293 (111)	M:38% F:62%	Depression, anxiety	CESD for depression; BAI for anxiety
**Grant& Keltner, (2011), Norris et al., (2011)**	Cross-sectional	519	Arab immigrant women living in metropolitan Detroit	Immigrants	Mixed Arabs (Iraqi (541, Lebanese (331%), born in the Middle East or Northern Africa(128%)	Mean age 4022 (65)	Female only	Depression	CESD
**Jamil et al. (2010)**	Pilot	285 (Iraqi refugees (n = 191), non-refugees (n = 94))	Psychiatric medical chart review	Refugees, non- refugees	Iraqi	Range: 18+	M: 465% F:355%	Major depression	Clinical assessment by psychiatrist in medical chart
**Wrobel, Farag and Haymes (2009)**	Cross-sectional	200	Community-based and religious groups in Dearborn, Michigan	Arab Americans (46%), refugees (8%), permanent residents (36%), visa (2%)	Mixed Arabs (largest proportion form Lebanon (42%), Iraq (33%), Yemen (11%), Palestine (101%)	Range: 60–92 Mean age 6911 (640)	M:465% F:535%	Depression	GDS
**Abu-Ras and Abou badr (2009)**	Cross-sectional	350	Community-based, health care, and religious organizations in California, Michigan, New York, New Jersey, and Washington, DC	Arab American	Mixed Arab ethnicities (22 countries), including Egyptian, Bahraini, Syrian, Tunisian, Algerian)	Range: 18 to 77 Mean age 344 (1171)	M: 45% F: 55%	Depression	CESD
**Hassouneh and Kulwicki (2007)**	Pilot	30	Personal networks, informational flyers	Immigrants	Palestine (334%), Iraqi (30%), Lebanese (233%), Syrian (67), Yemeni(33%) and African American (33%)	Range: 27–65 Mean age 38 (1042)	Female only	Depression, anxiety	BDI-II and CESD used for depression; BAI for anxiety
**Jamil (2007)**	Retrospective	116	Refugees’ health center medical records	Refugees	Iraqi	Range: 19–75 Mean age 518 (317)	M:604% F:396%	Depression, anxiety symptoms	HSC-25
**Jamil et al. (2002)**	Retrospective	375	Review medical charts of clients who sought mental health services at the Arab Community Center for Economic and Social Services (ACCESS) in Dearborn, Michigan, from 1998 to 1999	Refugees	Iraqi	< 17–40+	M: 621% F: 379%	Depressive disorders	Medical diagnosis by mental health professionals

**Legend**: NR (Not reported), F(Female), M(Male), NHIS (National Health Interview Survey)

Depression measures: CES-D (Center for Epidemiological Studies Depression Scale), CESD-SF (Center for Epidemiologic Studies Depression - Short Form), BDI-II (Beck Depression Inventory II), GDS (Geriatric Depression Scale), PHQ9 (patient Health Questionnaire-9), PHQ4 (Patient Health Questionnaire-4), HSC-25 (Hopkins Symptom Checklist 25 items).

Anxiety measures: SCARED (The Screen for Child Anxiety Relied Disorders), GAD-4 (Generalized Anxiety Disorders), DASS (Depression Anxiety Stress Scales), BAI (Beck Anxiety Inventory)

**Correct version of Table 2**

**Table 2:** Study and participants characteristics

Author, Year	Study Design	Sample Size	Sample Recruitment Source	Immigration Status	Ethnic Group	Age (years)	Gender	Mental Health Symptoms	Outcome Measurement
Abuelezem et al. (2022) and Abuelezem et al. (2021)	Cross-sectional	18, 072 Arab Americans and 5777 Iranian Americans	Electronic health records (EHRs) from a 2016 Northern California health plan cohort	Arab American	Ethnicities of Arab Americans from 22 countries, Iranian Americans	Range: 35- 84 Mean age for Arab Americans of both genders was 532	M:507% F:493%	Depression or Anxiety	Clinical diagnosis of anxiety or depression
Abuelezam and El-Sayed (2021)	Cross-sectional	354 Arab American and 3517 non-Arab/ non - Hispanic whites	Arab Community Center for Economic and Social Services (ACCESS) from the standalone 2013 Michigan Arab Behavioral Risk Factor Survey (MI ABRFS)	Arab American	NR	Range: 18- 40+	M:534% F:466%	Depression	Reported health professional depression diagnosis
Suleiman and Whitfield (2021)	Cross-sectional	142	Community (Arab Community Center for Economic and Social Services (ACCESS) facility in Dearborn, Michigan ACCESS	75% Immigrants	Mixed Arab ethnicity (Iraqi (35%), Lebanese (13%), Syrian (13%), Yemenite (13%), US- born (15%))	Range: 18+	M:30% W:70%	Depression	CESD
M’zah, Cardozo and Evans (2019)	Cross-sectional	25	Community organization supporting Syrian refugees in Metropolitan Atlanta	Refugees	Syrian	Range: 18 + Mean age 375 (94)	M: 376% F: 373%	Anxiety symptoms, and depression	HSC 25 used for anxiety, depression
Javanbakht et al. (2019)	Cross-sectional	157	Two primary care clinics in the Detroit area of Michigan	Refugees	Syrian	Range: 18– 65 Mean age: 3608 (1141)	M: 529% F:471%	Anxiety and depression	HSC-25
Javanbakht et al. (2018)	Cross-sectional	131	Children from two primary care clinics in the Detroit area of Michigan	Refugees’ children	Syrian	Range: 6- 17 years Mean age 1102 (332)	M: 595% F: 405%	Anxiety and depression	The SCARED instrument for anxiety, HSC-25 for depression
Aroian, Uddin and Pham (2015)	Cross-sectional	538	Researcher community network	Arab immigrants (447%) refugees (445%)	Mixed ethnicity - Iraq (437%), Lebanese (338%)	Range: 18 + Mean age 4024 (647)	Female only	Depression	CESD
Jaber et al. (2015)	Cross-sectional	98	The ACCESS Child and Adolescent Health Center and the Helping Youth Progress and Excel (HYPE) Recreation Center, Dearborn, Michigan	Arab immigrants (- generation immigrants (69%), first- generation (20%), third generation (10%))	Mixed ethnicity (mostly US born (79%), Saudi (5%), Yemen(5%), Lebanon(5%), Iraq(4%))	Range: 12 to 17 Mean age 154 (15)	M:57% F:43%	Depression	PHQ9
Kira et al. (2014)	Cross- ectional	399	Mental health clinic	Arab Americans (688%) and refugees (312%)	Mixed (Iraqi, (278%), Lebanese (278%), Yemenis (215%), other Arabs (56%)	Range: 18 to 76 Mean Age 66 (1145)	M:535% F:464%	Depression or anxiety	CESD, DASS for anxiety and depression
Tylor et al. (2014)	Cross-sectional	366	Local refugee resettlement agencies’ lists	Refugees, or on special immigrant visas	Iraqi	Range: 18+	M:60% F: 40%	Anxiety or depression	HSC-25 for anxiety, depression
Amer and Hovy (2012)	Cross-sectional	601	Email invitation to Arab American personal contacts	Arab American	Mixed Arab ethnicity (Palestinian (245%), Egyptian (198%), Lebanese(161 %), Syrian (68%), Iraqi(5%))	Range: 18- 81 Mean age 293 (111)	M:38% F:62%	Depression, anxiety	CESD for depression; BAI for anxiety
Grant& Keltner, (2011), Norris et al., (2011)	Cross-sectional	519	Arab immigrant women living in metropolitan Detroit	Immigrants	Mixed Arabs (Iraqi (541, Lebanese (331%), born in the Middle East or Northern Africa(128%)	Mean age 4022 (65)	Female only	Depression	CESD
Jamil et al. (2010)	Pilot	285 (Iraqi refugees (*n* = 191), non-refugees (*n* = 94))	Psychiatric medical chart review	Refugees, non- refugees	Iraqi	Range: 18+	M: 465% F:355%	Major depression	Clinical assessment by psychiatrist in medical chart
Abu-Ras and Abou badr (2009)	Cross-sectional	350	Community-based, health care, and religious organizations in California, Michigan, New York, New Jersey, and Washington, DC	Arab American	Mixed Arab ethnicities (22 countries), including Egyptian, Bahraini, Syrian, Tunisian, Algerian)	Range: 18 to 77 Mean age 344 (1171)	M: 45% F: 55%	Depression	CESD
Hassouneh and Kulwicki (2007)	Pilot	30	Personal networks, informational flyers	Immigrants	Palestine (334%), Iraqi (30%), Lebanese (233%), Syrian (67), Yemeni(33%) and African American (33%)	Range: 27- 65 Mean age 38 (1042)	Female only	Depression, anxiety	BDI-II and CESD used for depression; BAI for anxiety
Jamil (2007)	Retrospective	116	Refugees’ health center medical records	Refugees	Iraqi	Range: 19- 75 Mean age 518 (317)	M:604% F:396%	Depression, anxiety symptoms	HSC-25
Jamil et al. (2002)	Retrospective	375	Review medical charts of clients who sought mental health services at the Arab Community Center for Economic and Social Services (ACCESS) in Dearborn, Michigan, from 1998 to 1999	Refugees	Iraqi	18–40+	M: 621% F: 379%	Depressive disorders	Medical diagnosis by mental health professionals

Legend: NR (Not reported), F(Female), M(Male), NHIS (National Health Interview Survey). Depression measures: CES-D (Center for Epidemiological Studies Depression Scale), CESD-SF (Center for Epidemiologic Studies Depression - Short Form), BDI-II (Beck Depression Inventory II), GDS (Geriatric Depression Scale), PHQ9(patient Health Questionnaire-9), PHQ4 (Patient Health Questionnaire-4), HSC-25 (Hopkins Symptom Checklist 25 items).Anxiety measures: SCARED (The Screen for Child Anxiety Relied Disorders), GAD-4(Generalized Anxiety Disorders), DASS (Depression Anxiety Stress Scales), BAI (Beck Anxiety Inventory)

**Incorrect version of Table 3**

**Table 3:** Subgroup analyses of the prevalence of depression and anxiety among arab individuals

Subgroup Comparison	Pooled Prevalence of Depression				Pooled Prevalence of Anxiety			
	# of Studies	Pooled Prevalence	95% CI		# of Studies	Pooled Prevalence	95% CI	
			Lower limit	Upper limit			Lower limit	Upper limit
**Gender Composition**								
Women Only	3	0.45	0.29	0.61	1	0.67	0.49	0.81
Mixed	14	0.42	0.35	0.50	9	0.48	0.28	0.68
Total	17	0.43	0.36	0.50	10	0.49	0.30	0.68
**Immigration Status**								
Refugees	6	0.50	0.23	0.78	4	0.60	0.39	0.81
Immigrants	4	0.41	0.08	0.75	2	0.11	0.10	0.11
Arab American	5	0.27	0.06	0.78	1	0.47	0.43	0.51
Mixed	2	0.71	0.68	0.74	3	0.44	0.02	0.85
Total	17	0.43	0.36	0.50	10	0.49	0.30	0.68
**Ethnicity**								
Iraqi or Syrian	5	0.60	0.46	0.74	4	0.62	0.43	0.80
Mixed	12	0.36	0.29	0.44	6	0.41	0.19	0.63
Total	17	0.43	0.36	0.50	10	0.49	0.30	0.68
**Study Type**								
Cross-sectional	13	0.37	0.30	0.45	8	0.44	0.23	0.64
Pilot	2	0.69	0.63	0.75	1	0.67	0.49	0.81
Retrospective	2	0.60	0.56	0.64	1	0.80	0.72	0.86
Total	17	0.43	0.36	0.50	10	0.49	0.30	0.68
**Study Quality**								
High	4	0.30	.09	0.51	3	0.36	0.06	0.66
Medium	7	0.55	0.30	0.81	6	0.53	0.25	0.82
Low	6	0.38	0.08	0.68	1	0.67	0.49	0.81
Total	17	0.43	0.36	0.50	10	0.49	0.30	0.68

Abbreviations: df; degree of freedom

**Correct version of Table 3**

**Table 3:** Subgroup analyses of the prevalence of depression and anxiety among arab individuals

Subgroup Comparison	Pooled Prevalence of Depression				Pooled Prevalence of Anxiety			
	# of Studies	Pooled Prevalence	95% CI		# of Studies	Pooled Prevalence	95% CI	
			Lower limit	Upper limit			Lower limit	Upper limit
**Gender Composition**								
Women Only	3	0.47	033	0.60	1	0.53	0.29	0.77
Mixed	14	0.49	0.32	0.66	9	0.97	0.83	0.99
Total	17	0.48	0.34	0.63	10	0.58	0.33	0.83
**Immigration Status**								
Refugees	6	0.52	0.37	0.68	4	0.61	0.42	0.81
Immigrants	4	0.42	0.11	0.74	2	0.12	0.11	0.12
Arab American	5	0.41	0.25	0.58	1	0.47	0.43	0.51
Mixed	2	0.71	0.68	0.74	3	0.64	0.61	0.67
Total	17	0.48	0.34	0.63	10	0.58	0.33	0.83
**Ethnicity**								
Iraqi or Syrian	5	0.52	0.33	0.72	4	0.56	0.46	0.68
Mixed	12	0.46	0.28	0.64	6	0.57	0.17	0.97
Total	17	0.48	0.34	0.63	10	0.58	0.33	0.83
**Study Type**								
Cross-sectional	13	0.48	0.31	0.65	8	0.50	0.25	0.74
Pilot	2	0.29	0.33	0.35	1	0.97	0.83	0.99
Retrospective	2	0.60	0.56	0.64	1	0.80	0.72	0.86
Total	17	0.48	0.34	0.63	10	0.58	0.33	0.83
**Study Quality**								
High	4	0.32	0.11	0.52	3	0.36	0.07	0.66
Medium	7	0.57	0.40	0.75	6	0.65	0.49	0.81
Low	6	0.51	0.44	0.58	1	0.93	0.87	0.99
Total	17	0.48	0.34	0.63	10	0.58	0.33	0.83

Abbreviations: df; degree of freedom

**Incorrect version of Table 4**

**Table 4:** Results of the random effects meta-regression of the studies and demographic moderators for the prevalence depression and anxiety

	Depression					Anxiety				
Moderator	Coefficient	Standard error	t-value	p-value	95% CI	Coefficient	Standard error	t-value	p-value	95% CI
Total Sample Size	− .0025	.0001	-1.83	0.087	.0005, 3.71	-0.0002	0.00014	-1.76	.117	-0.0005, 7.54
Publication Year	-0.022	0.008	-2.55	*0.02	− .040, − .004	-0.014	0.016	0.087	0.410	-0.529,0.023
Mean age of Arab Individuals	− .0085	0.005	-1.55	0.152	-0.020, 0.0036	− .011	0.002	-4.10	**0.005	-0.018, -0.0050
Proportion of Female Arabs	− .00026	0.003	0.78	0.453	-0.0048, 0.01003	0.129	0.351	0.37	0.728	-0.775, 1.034
Proportion of Married Arabs	-0.0627	0.446	-0.14	0.892	-0.015, 0.966	-0.597	0.405	-0.15	0.890	-1.185, 1.065
Proportion of Arab with lower education (high school or less)	− .0073	0.005	-1.43	0.191	-0.018, 0.0045	0.025	0.455	0.06	0.958	-1.423, 1.475
Ethnicity Being Iraqi or Syrian	0.232	0.126	1.84	0.086	-0.036, 0.5021	0.209	0.161	1.30	0.230	-0.162, 0.582

Abbreviations: CI; Confidence Interval

**Correct version of Table 4**

**Table 4:** Results of the random effects meta-regression of the studies and demographic moderators for the prevalence depression and anxiety

Moderator	Depression					Anxiety				
	Coefficient	Standard error	t-value	*p*-value	95% CI	Coefficient	Standard error	t-value	*p*-value	95% CI
Total Sample Size	− 0.0025	0.00012	-1.83	0.087	0.0005, 3.71	-0.00024	0.00014	-1.76	0.117	-0.00056, 0.0044
Publication Year	-0.022	0.0089	-2.55	*0.02	− 0.040, − 0.004	-0.0146	0.0167	0.087	0.410	-0.529, 0.0239
Mean age of Arab Individuals	− 0.0085	0.0054	-1.55	0.152	-0.0206, 0.0036	− 0.0118	0.0028	-4.10	**0.005	-0.0186, -0.0050
Proportion of Female Arabs	− 0.00026	0.0033	0.78	0.453	-0.0048, 0.010039	0.1295	0.3517	0.37	0.728	-0.775, 1.034
Proportion of Married Arabs	-0.0627	0.4461	-0.14	0.892	-0.0156, 0.9660	-0.597	0.4054	-0.15	0.890	-1.185, 1.065
Proportion of Arab with lower education (high school or less)	− 0.0073	0.0051	-1.43	0.191	-0.0189, 0.0045	0.0258	0.4552	0.06	0.958	-1.423, 1.475
Ethnicity Being Iraqi or Syrian	0.232	0.1265	1.84	0.086	-0.0369, 0.5021	0.209	0.1614	1.30	0.230	-0.162, 0.582

Abbreviations: CI; Confidence Interval

**Incorrect version of Table 5**

**Table 5:** Methodological quality of the included studies

Author (year)	1. Selection Bias: Comparable Study Groups or Statistical Adjustment	2. Measurement Bias: Valid and Reliable Measurement of the Exposure	3. Attrition Bias (for Cohort Study): Similar Drop- out Rates in Both Case	4. Loss Bias (Cohort Study): no Systematic Differences between Completers and Drop- outs	5. External Validity: Consecutive or Randomized Selection	6. External Validity: Representative Sample	Overall Evaluation of Methodological Quality
Patel et al. (2022)	**+**	**+**			**+**	**+**	**High**
Abuelezem et al. (2022) & Abuelezem et al. (2021)	**+**	**+**			**-**	**+**	**High**
Abuelezam & El- Sayed (2021)	**0**	**+**			**+**	**+**	**High**
Suleiman & Whitfield (2021)	**+**	**+**			**-**	**-**	**Medium**
Arnetz et al. (2020)	**0**	**+**			**-**	**-**	**Low**
Javanbakht et al. (2020)	**+**	**+**			**-**	**-**	**Medium**
Kader et al. (2020)	**+**	**+**			**-**	**0**	**Medium**
M’zah, Cardozo & Evans (2019)	**0**	**+**			**-**	**-**	**Low**
Javanbakht et al. (2019)	**+**	**+**			**-**	**-**	**Medium**
Javanbakht et al. (2018)	**+**	**+**			**-**	**-**	**Medium**
Pampati et al. (2018)	**+**	**+**			**-**	**0**	**Medium**
Lemaster et al. (2018)	**+**	**+**	**+**	**0**	**+**	**+**	**High**
Arion, Uddin & Blbas (2017)	**0**	**+**	**+**	**0**	**0**	**+**	**Medium**
Aroian, Uddin & Pham (2015)	**0**	**+**			**0**	**+**	**Medium**
Jaber et al. (2015)	**0**	**+**			**-**	**0**	**Low**
Kira et al. (2014)	**+**	**+**			**-**	**-**	**Medium**
Tylor et al. (2014)	**+**	**+**			**+**	**+**	**High**
Amer & Hovy (2012)	**+**	**+**			**-**	**+**	**High**
Grant& Keltner (2011) & Norris et al. (2011)	**0**	**+**			**-**	**0**	**Low**
Jamil (2010)	**+**	**+**			**-**	**-**	**Medium**
Hassouneh & Kulwicki (2007)	**0**	**+**			**-**	**-**	**Low**
Jamil, 2002	**-**	**+**			**-**	**-**	**Low**

(*) Codes used are as follows: + = criterion met, - = criterion not met, 0 = missing information or unclear criterion

**Correct version of Table 5**

**Table 5:** Methodological quality of the included studies

Author (year).	1. Selection Bias: Comparable Study Groups or Statistical Adjustment	2. Measurement Bias: Valid and Reliable Measurement of the Exposure	5. External Validity: Consecutive or Randomized Selection	6. External Validity: Representative Sample	Overall Evaluation of Methodological Quality
Abuelezem et al. (2022) & Abuelezem et al. (2021)	**+**	**+**	**-**	**+**	**High**
Abuelezam & El- Sayed (2021)	**0**	**+**	**+**	**+**	**High**
Suleiman & Whitfield (2021)	**+**	**+**	**-**	**-**	**Medium**
M’zah, Cardozo & Evans (2019)	**0**	**+**	**-**	**-**	**Low**
Javanbakht et al. (2019)	**+**	**+**	**-**	**-**	**Medium**
Javanbakht et al. (2018)	**+**	**+**	**-**	**-**	**Medium**
Aroian, Uddin & Pham (2015)	**0**	**+**	**0**	**+**	**Medium**
Jaber et al. (2015)	**0**	**+**	**-**	**0**	**Low**
Kira et al. (2014)	**+**	**+**	**-**	**-**	**Medium**
Tylor et al. (2014)	**+**	**+**	**+**	**+**	**High**
Amer & Hovy (2012)	**+**	**+**	**-**	**+**	**High**
Grant& Keltner (2011) & Norris et al. (2011)	**0**	**+**	**-**	**0**	**Low**
Jamil (2010)	**+**	**+**	**-**	**-**	**Medium**
Abu-Ras and Abou badr (2009)	**-**	**+**	**-**	**+**	**Medium**
Hassouneh & Kulwicki (2007)	**0**	**+**	**-**	**-**	**Low**
Jamil, 2007	**-**	**+**	**-**	**-**	**Low**
Jamil, 2002	**-**	**+**	**-**	**-**	**Low**

(*) Codes used are as follows: + = criterion met, - = criterion not met, 0 = missing information or unclear criterion

The original article has been corrected.

## Electronic Supplementary Material

Below is the link to the electronic supplementary material.

Supplementary Material 1

