# Supplementary material for: Depression and Anxiety Among Arab Individuals in the United States: A Meta-analysis
Source: J Immigr Minor Health. 2024 Nov 27;27(2):329–50. doi: 10.1007/s10903-024-01648-9 (PMC11903594; doi:10.1007/s10903-024-01648-9)
Supplement: Supplementary file 1 — Supplementary file1 (DOCX 43 KB) [file 10903_2024_1648_MOESM1_ESM.docx]

# Supplement 1: Search Strategies.

| **Database: Psych INFO, Psych Articles, Sociological Abstract**  **Date**: 4/5/2022 | |  |
| --- | --- | --- |
| **Set #** | **Search Terms/ Keyword** | **Results (Ns)** |
| 1 | (("arabs"[MeSH Terms] OR "arab" [tiab] OR "arabs" [tiab] OR Arabic [tiab]) AND ("emigrants and immigrants"[MeSH Terms] OR "immigrants" [tiab] OR" migrants" [tiab] OR "emigration and immigration"[MeSH Terms] OR "emigration" [tiab] OR "immigration" [tiab] OR "immigrate" [tiab] OR "transients and migrants"[MeSH Terms] OR "refugees"[MeSH Terms] OR refugees [tiab] OR refugee [tiab] OR asylum [tiab] OR ("asylum"[tiab] AND "seekers"[tiab]) OR "asylum seekers"[tiab] ) ) OR "Arab Americans"  [tiab] OR "Arab American" [tiab] | 1714 |
| 2 | "depressive disorder"[MeSH Terms] OR "depression"[MeSH Terms] OR “depression” [tiab] OR “depressions” [tiab] OR "depressive disorder"[tiab] OR “depressive disorders” [tiab] OR "anxiety"[MeSH Terms] OR “anxiety” [tiab] OR "anxieties"[tiab] OR Anxiety Disorders [mh] OR "mental health"[MeSH Terms] OR "mental health"[tiab] OR "mental disorders"[MeSH Terms] OR “mental disorders” [tiab] OR “mental disorder” [tiab] | 2,668,332 |
| 3  Combined | #1 AND #2 | 482 |

| **Database: Embase**  **Date**: 4/5/2022 | |  |
| --- | --- | --- |
| **Set #** | **Search Terms/ Keyword** | **Results (Ns)** |
| 1 | ('arab'/exp OR arab OR Arabic OR arabs) AND ('immigrants'/exp OR immigrant OR immigrants OR 'emigrant'/exp OR emigrant IR emigrants OR 'asylum seekers'/exp OR 'asylum seekers' OR 'asylum seekers' OR 's]asylum') OR 'arab american' OR 'arab americans' | 1462 |
| 2 | 'depression'/exp OR depression OR depressive OR 'anxiety disorder'/exp OR 'anxiety'/exp OR anxiety OR anxieties OR 'mental health'/exp OR 'mental health' OR 'mental disease'/exp OR 'mental disease' OR 'mental disorder'/exp OR 'mental disorder' OR 'mental disorders'/exp OR 'mental disorders' | 6,349,798 |
| 3  Combined | #1 AND #2 | 493 |

| **Database: Web of Science**  **Date**: 4/5/2021 | |  |
| --- | --- | --- |
| **Set #** | **Search Terms/ Keyword** | **Results (Ns)** |
| 1 | (("arabs"[MeSH Terms] OR arab OR arabs OR Arabic) AND ("emigrants and immigrants"[MeSH Terms] OR immigrants OR emigrants OR "emigration and immigration"[MeSH Terms] OR "emigration" OR "immigration" OR "immigrate" OR "transients and migrants"[MeSH Terms] OR "refugees"[MeSH Terms] OR refugees OR refugee OR asylum OR "asylum seekers") ) OR "Arab Americans" OR "Arab American" | 2738 |
| 2 | "depressive disorder"[MeSH Terms] OR "depression"[MeSH Terms] OR “depression” OR “depressions” OR "depressive disorder" OR “depressive disorders” OR "anxiety"[MeSH Terms] OR “anxiety” OR "anxieties" OR Anxiety Disorders [mh] OR "mental health"[MeSH Terms] OR "mental health" OR "mental disorders"[MeSH Terms] OR “mental disorders” OR “mental disorder” | 3,201,420 |
| 3  Combined | #1 AND #2 | 457 |

| **Database:** PROQUEST PsycArticles, PsycInfo, PsycTests, GenderWatch, PAIS Index, PTSDpubs, Sociological Abstracts, Social Services Abstracts  **Date**: 4/5/2022 | |  |
| --- | --- | --- |
| **Set #** | **Search Terms/ Keyword** | **Results (Ns)** |
| 1 | ab((( (Arabs OR Arab OR Arabic) AND (immigrants OR immigrant OR emigrants OR emigration OR immigration OR immigrate OR refugees OR refugee OR asylum OR "asylum seekers") ) OR "Arab Americans" OR "Arab American”) AND (depression OR "depressive disorder" OR "depressive disorders" OR anxiety OR anxieties OR "anxiety  disorders" OR "anxiety disorder" OR "mental health" OR "mental disorder" OR "mental disorders" OR "post-traumatic stress disorder" OR PTSD OR trauma OR stress) ) | 502 |
| 2 | ti((((Arabs OR Arab OR Arabic) AND (immigrants OR immigrant OR emigrants OR emigration OR immigration OR immigrate OR refugees OR refugee OR asylum OR "asylum seekers")) OR "Arab Americans" OR "Arab American") AND (depression OR "depressive disorder" OR "depressive disorders" OR anxiety OR anxieties OR "anxiety disorders" OR "anxiety disorder" OR "mental health" OR "mental disorder" OR "mental disorders")) OR ab((((Arabs OR Arab OR Arabic) AND (immigrants OR immigrant OR emigrants OR emigration OR immigration OR immigrate OR refugees OR refugee OR asylum OR "asylum seekers")) OR "Arab Americans" OR "Arab American") AND (depression OR "depressive disorder" OR "depressive disorders" OR anxiety OR anxieties OR "anxiety disorders" OR "anxiety disorder" OR "mental health" OR "mental disorder" OR "mental  disorders")) | 505 |
| 3  Combined | Limited to “Peer Reviewed”  #1 AND #2 | 432 |

| **Database: CINAHL Date**: 4/5/2022 | |  |
| --- | --- | --- |
| **Set #** | **Search Terms/ Keyword** | **Results (Ns)** |
| 1 | (((MH "Arabs") OR Arab OR Arabic OR Arabs) AND (MH "Refugees" OR MH "Immigrants+" OR refugees OR refugees OR immigrants OR immigrants OR immigration OR emigrants OR emigrant OR emigration OR asylum) ) OR “Arab American” OR “Arab Americans” | 1,918 |
| 2 | (MH “Depression”) OR depression OR depressive OR (MH “Anxiety+) OR Anxiety OR Anxieties OR (MH “Anxiety Disorders” OR (MH “mental Disorders”) OR “mental disorder” OR “mental disorders” OR (MH “Mental Health”) OR “mental health” | 478,684 |
| 4  Combined | Limited to Academic Journals #1 AND #2 | 420 |

**Supplement 2: Description of Excluded Studies**

| **Author, Year** | **Title** | **Key Findings** | **Reason for Exclusion** |
| --- | --- | --- | --- |
| Seff et al., 2021 | Psychosocial Well-Being, Mental Health, and Available Supports in an Arab Enclave: Exploring Outcomes for Foreign- Born and U.S.-Born Adolescents | No statistically significant differences in immigration were observed between the foreign-born and U.S.- born groups. | Mixed method study. |
| Alkaid Albqoor et al., 2020 | Serious psychological distress: A national study of Middle Eastern immigrants | Serious psychological distress rate was the largest for Middle Eastern immigrants (5.99%) between 2006 and 2010. | The researchers focused on psychological distress not anxiety or depression. |
| Javanbakht et al., 2020 | Perceived health, adversity, and posttraumatic stress disorder in Syrian and Iraqi refugees perceived health, adversity, and posttraumatic stress disorder in Syrian and Iraqi refugees | Self-reported rating of violence, armed conflict, and flight related adverse experiences were significantly associated with perceived well-being among refugees. | No prevalence investigated. |
| Amin & Driver, 2019 | Sex differences, duration of stay in the United States, and serious psychological distress: The case of Middle Eastern immigrants in the United States | There was a significant association between the length of immigration and self-reported serious psychological distress symptoms among Middle Eastern immigrant women. | The study focused on psychological distress not anxiety or depression. |
| Krohner et al., 2019 | Somatic and depressive symptoms in Arab American women: The influence of religion on social and emotional risk factors | No abstract available. | Full text is not accessible. |
| Uygun et al., 2018 | Factors associated with PTSD in a group of Syrian refugees who applied to immigrant/refugee mental health special branch outpatient clinic | No abstract available. | Full text is not accessible. |
| Wilson &Thayer, 2018 | Impact of acculturation on depression, perceived stress, and self-esteem in young Middle Eastern  American adults | Integrated middle Eastern Arabs showed less stress or depression than assimilated Arabs. | No data on prevalence was provided. |
| Assari and Lankarni., 2017 | Discrimination and Psychological Distress: Gender Differences among Arab Americans | Discrimination was found to have a positive association with psychological distress among Arab Americans | The study focused on psychological distress not anxiety or depression. |
| Ikizler and Szymanski, 2017 | Discrimination, religious and cultural factors, and Middle Eastern/Arab Americans' psychological distress | Among Arab Americans, more discrimination, increased family connectedness, the interaction of discrimination and religiosity, and the interaction of discrimination and family connectedness were identified as unique predictors of psychological distress. | The study focused on psychological distress not anxiety or depression. |
| Abuelezam et al., 2017 | Depression Among Arab American and Arab Immigrant Women in the United States | Arab American women in the U.S. were more likely to experience a multitude of stresses, such as immigration stress and intimate partner abuse, which increased their chances of developing depression. | This was a review article. |
| Pampati et al., 2017 | Assessing the influence of migration status on self- rated health outcomes: a cross sectional study of Arab American in Michigan | No abstract available. | Full text is not accessible. |
| Samri, 2016 | Cross-border ties and Arab American mental health | Cross-border attitudes and social ties were associated with larger odds of psychological distress for first generation Arab Americans. | The study focused on psychological distress not anxiety or depression |
| Arnetz et al., 2013 | Resilience as a protective factor against the development of psychopathology among refugees | Resilience was found to be associated with lower levels of trauma-related psychological distress among Arab immigrants. | The study focused on mental disorders other than anxiety or depression. |
| Jamil et al., 2009 | The prevalence of self- reported chronic conditions among Arab, Chaldean, and African Americans in Southeast Michigan | Chaldeans were less likely to report having one more chronic condition compared to whites. | No access to full text. |
| Norris and Aronia., 2008 | Avoidance symptoms and assessment of posttraumatic stress disorder in Arab immigrant women | Arab immigrant women reported experiencing more PTSD. | The study focused on mental disorders other than anxiety or depression. |
| Jamil et a., 2008 | Factors associated with self-reported depression in Arab, Chaldean, and African Americans | The largest rate of depression was found in Arab American participants; African Americans and Chaldeans showed lower rates. | No access to full text. |
| Khuwaja et al., 2007 | Pakistani Ismaili Muslim Adolescent Females Living in the United States of America: Stresses Associated with the Process of Adaptation to U.S. Culture | Longer periods of stay in the United States, young age at migration, and speaking more English were associated with lower sociopsychological stress scores among Pakistani Muslims. | The researchers studied a population which is not Arabic. |
| Kira et al., 2007 | Health issues in the Arab American community. The physical and mental status of Iraqi refugees and its etiology | No abstract available. | Full text is not accessible. |
| Aboumediene, Farrag & Dakroub, 2005. | Depression and medical health complaints in a group of Arab-American women | No abstract available. | Full text is not accessible. |
| Amer & Hovy, 2005 | Examination of the impact of acculturation, stress, and religiosity on mental health variables for second-generation Arab Americans | No abstract available. | Full text is not accessible. |
